# Comparison of surgical and endovascular left subclavian artery revascularization during thoracic aortic endovascular repair: a systematic review and meta-analysis

**DOI:** 10.3389/fcvm.2023.1274629

**Published:** 2023-11-02

**Authors:** Feng Lin, Zhipeng He, Junpeng Gao, Xiaoci Huang, Haoran Wang, Long Han, Xingyang Zhu, Yanqing Zhan, Wenbin Wang

**Affiliations:** ^1^ Department of General Surgery, The First Affiliated Hospital of Anhui Medical University, Hefei, China; ^2^ Department of General Surgery, Anhui Public Health Clinical Center, Hefei, China; ^3^ Department of General Surgery, The Second Affiliated Hospital of Nanchang University, Nanchang, China; ^4^ Department of Anaesthesiology, The Second Affiliated Hospital of Anhui Medical University, Hefei, China; ^5^ Department of Vascular Surgery, The First Affiliated Hospital of Wannan Medical College, Wuhu, China; ^6^ Department of General Surgery, The Second Affiliated Hospital of Anhui Medical University, Hefei, China; ^7^ Department of Vascular Surgery, The Second Affiliated Hospital of Anhui Medical University, Hefei, China

**Keywords:** thoracic endovascular aortic repair, left subclavian artery, revascularization, stroke, meta-analysis

## Abstract

**Objective:**

The purpose of this systematic review and meta-analysis was to incorporate data from the latest clinical studies and compare the safety and efficacy of surgical left subclavian artery (LSA) revascularization and endovascular LSA revascularization during thoracic endovascular aortic repair (TEVAR).

**Methods:**

This study was performed in accordance with the Preferred Reporting Items for Systematic Reviews and Meta-Analyses (PRISMA) guidelines and was registered with the PROSPERO database on 16 April 2023 (CRD42023414579). The Embase, MEDLINE (PubMed), and the Cochrane Library databases were searched from January 2000 to May 2023.

**Results:**

A total of 14 retrospective cohort studies with a total of 1,695 patients, were included for review. The peri-operative stroke rates of the surgical and endovascular LSA revascularization groups were 3.8% and 2.6%, respectively (*P* = 0.97). The peri-operative technical success rates for the surgical and endovascular LSA revascularization groups were 95.6% and 93.0%, respectively (*P* = 0.24). The peri-operative spinal cord ischemia rates were 1.6% (*n* = 18) and 1.9% (*n* = 7) in the surgical and endovascular LSA revascularization groups, respectively (*P* = 0.90). The peri-operative type Ⅰ endoleak rates for the surgical and endovascular LSA revascularization groups were 6.6% and 23.2%, respectively (*P* = 0.25). The subgroup analysis showed that the incidence of peri-operative type I endoleak in the parallel stent group was significantly higher than that in the surgical LSA revascularization group (*P* < 0.0001). The peri-operative left upper limb ischemia rates for the surgical and endovascular LSA revascularization groups were 1.2% and 0.6%, respectively (*P* = 0.96). The peri-operative mortality rates of the surgical and endovascular LSA revascularization groups were 2.0% and 2.0%, respectively (*P* = 0.88).

**Conclusion:**

There was no significant difference in the terms of short-term outcomes when comparing the two revascularization techniques. The quality of evidence assessed by GRADE scale was low to very-low. Surgical and endovascular LSA revascularization during TEVAR were both safe and effective. Compared with surgical LSA revascularization techniques, parallel stent revascularization of LSA significantly increased the rate of type I endoleak.

## Introduction

In 1994, Duke et al. first implemented thoracic aortic endovascular repair (TEVAR), which has since been widely used in clinical practice; its safety and efficacy has also been widely recognized (1, 2). To fully exclude aortic lesions, it is generally recommended that the proximal sealing zone be at least 2 cm, which leads to the need to cover the left subclavian artery (LSA) in some patients (3). However, numerous studies have shown that LSA coverage was associated with the risk of stroke, spinal cord ischemia (SCI), and upper limb ischemia, suggesting the need for revascularization of the LSA during TEVAR (4, 5, 6).

The Society for Vascular Surgery (SVS) 2009 Practice Guidelines recommend routine preoperative LSA revascularization during TEVAR where the proximal seal necessitates coverage of the LSA (GRADE 2, level C). In patients where their anatomy affected critical organ perfusion, routine preoperative LSA revascularization was strongly recommended despite the very low quality of evidence (GRADE 1, level C) (7). However, the 2020 SVS Practice Guidelines state that preoperative or concurrent LSA revascularization is necessary for elective TEVAR (GRADE 1, level B) (8). Traditionally, LSA revascularization was achieved through surgical revascularization of the left carotid artery to LSA bypass or LSA to left carotid artery transposition. Protack et al. included 282 patients undergoing TEVAR and surgical LSA revascularization in a single center retrospective cohort study; the results of the 16-year study showed a 98% primary patency rate at 5 years, low morbidity, and the infrequent need for re-intervention (4). Currently, commonly used endovascular LSA revascularization techniques include the parallel stent technique (chimney, sandwich, and periscope technique), *in vitro* fenestration technique, *in situ* fenestration technique, and branched arch endografts (9). Thanks to the continuous research of enterprises and medical centers all over the world, a number of new endovascular devices suitable for a variety of technologies have been entered into clinical research, resulting in rapid progress and promising breakthroughs.

At present, there are no large sample randomized controlled studies or clinical guidelines to indicate which LSA revascularization technique is superior. Therefore, the purpose of this systematic review and meta-analysis was to incorporate the latest clinical studies and compare the safety and efficacy of surgical LSA revascularization and endovascular LSA revascularization during TEVAR.

## Materials and methods

This analysis was performed in accordance with the Preferred Reporting Items for Systematic Reviews and Meta-Analyses (PRISMA) guidelines and was registered with the PROSPERO database on April 16, 2023 (CRD42023414579) (10).

### Inclusion and exclusion criteria

Comparative studies [including observational studies and randomized controlled trials (RCTs)] comparing surgical LSA revascularization and endovascular LSA revascularization in TEVAR were included. We excluded studies that: (1) published in non-English language journals; (2) where the number of individual cases was less than 20; (3) studies that only included *in vitro* and animal experiments; (4) studies where double or triple branch revascularization was performed simultaneously; (5) the data were insufficient for statistical analysis; and (6) abstracts, case reports, letters, and single arm studies.

### Search methodology and data extraction

The Embase, MEDLINE (PubMed), and Cochrane Library databases were searched from January 2000 to May 2023. The detailed retrieval of the database process is visible in the Supplementary Appendix S1. Two researchers (F.L. and J.G.) independently reviewed the titles and abstracts of all identified literature. Disagreements were resolved through discussions with the third author (W.W.). The reference lists of included relevant studies were also searched.

Two authors (F.L. and Z.H.) independently extracted data from the included studies using predefined standardized data extraction tables. Extracted data included first author, country of origin, year of publication, study design, study period, number of participants, basic demographics, aortic pathology, LSA revascularization procedures, and major peri-operative outcomes. The primary outcome was peri-operative stroke. Peri-operative SCI, peri-operative type I endoleak, peri-operative mortality, peri-operative technical success, and peri-operative left upper limb ischemia comprised the secondary outcomes. The peri-operative period was defined as the in-hospital period or 30 days. Discrepancies between authors were resolved by consensus.

### Quality assessment

The risk of bias in individual studies was assessed using the Cochrane Bias Risk tool for RCTs and ROBINS-I tool for observational studies (11, 12). The Grading of Recommendations Assessment, Development, and Evaluation (GRADE) system was used to rate the quality of evidence and strength of each relevant outcome identified.

### Statistical analysis

Review Manager software (RevMan Version 5.4; Nordic Cochrane Centre, Copenhagen, Denmark) was used to summarize the data included in the meta-analysis. Relative risks (RRs) with 95% confidence intervals (CIs) were calculated to generate forest plots and to express differences for dichotomous outcomes. The *I*^2^ statistic was used to examine heterogeneity across the studies. Studies with an *I*^2 ^> 50% were considered to have significant heterogeneity and random effects models were used to pool the results. Otherwise, a fixed effect model would be applied. A *P* value <0.05 was considered statistically significant. When significant clinical heterogeneity existed, subgroup analysis or sensitivity analysis was performed.

## Results

### Study characteristics and risk of bias

**Figure 1:**
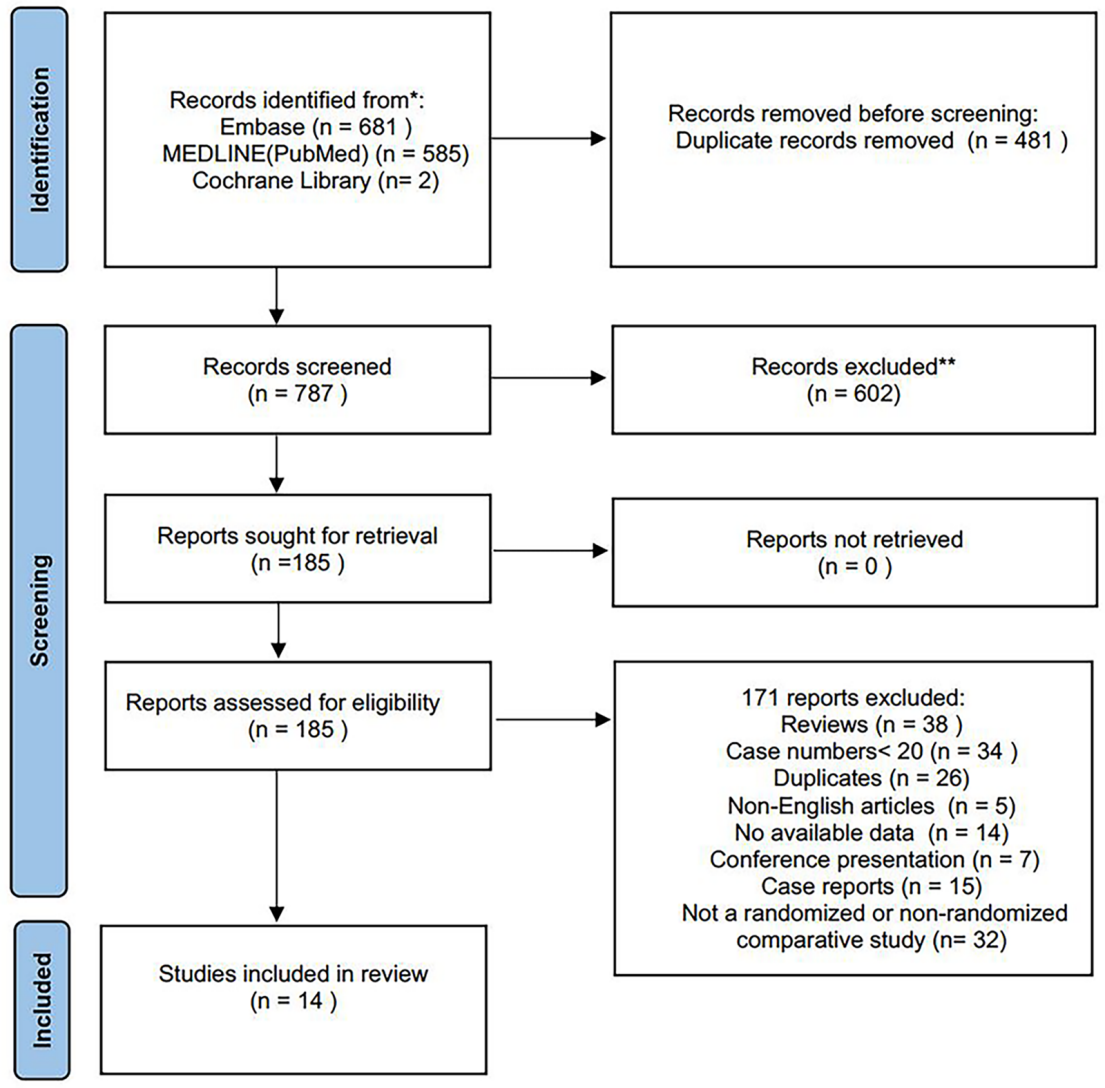
Study flow diagram according to the preferred reporting items for systematic reviews and meta-analyses (PRISMA) statement.

Of 1,268 records identified, 14 studies (retrospective cohort studies) with 1,695 patients were included in this systematic review (13–26). The PRISMA flow diagram is shown in Figure 1. No RCTs were identified and all included studies were published between 2017 and 2023. The included studies were geographically dispersed, with four from the United States, four from Europe, and six from China. The sample size ranged from 30 to 837 patients. Follow-up ranged from 13 to 36 months. Of 1,695 patients who underwent TEVAR and LSA revascularization, 1,204 underwent surgical LSA revascularization and 491 underwent endovascular LSA revascularization. Further study characteristics were summarized in Tables 1, 2.

**Table 1:** Characteristics of the included studies.

Studies (ER/SR)	Study period	Number of patients	Number of ER	Number of SR	Age,years, mean ± SD	Men, No.(%)	DM, No.(%)	Hypertension, No.(%)	Follow-up (month)
Bradshaw 2016	2007–2014	54	33	21	/	/	/	/	24
Zhao 2017	2010–2016	57	31	26	55.4 ± 11.3/52.6 ± 12.7	25 (80.6)/24 (92.3)	2 (6.5)/1 (3.8)	20 (64.5)/21 (80.8)	27/30
Piffaretti 2018	2010–2017	73	31	42	70 ± 13 /66 ± 12	21 (67.7) /35 (83.3)	4 (13)/6 (14)	27 (87)/34 (80)	24
Xiang 2018	2011–2017	38	24	14	54.8 ± 13.1 / 53.1 ± 12.9	21 (87.5)/13 (92.9)	/	19 (79.2)/14 (100)	26
Wang 2019	2015–2018	49	17	32	56.0 ± 10.6/52.4 ± 12.3	14 (82.4)/28 (87.5)	/	17 (100)/32 (100)	13
Konstantinou 2020	2012–2018	36	19	17	65.8 ± 2 /68.0 ± 3	11 (58)/14 (82)	2 (10.5)/0 (0)	18 (94.7)/13 (76.5)	15/17
D’Oria 2020	2013–2018	837	116	721	67 ± 12/64 ± 13	75 (65)/468 (65)	20 (17.0)/91 (12.6)	105 (91)/661 (92)	/
Johnson 2020	2013–2018	53	18	35	/	13 (72.0)/26 (74.3)	0 (0)/3 (8.6)	16 (88.9)/32 (91.4)	/
Ramdon 2020	2011–2017	81	17	64	68 ± 13/68 ± 11	10 (59) /43 (67)	1 (6)/10 (16)	15 (88)/55 (86)	8 /15
Dueppers 2021	2009–2020	48	24	24	75/71	/	0 (0)/3 (12.5)	19 (79) /17 (71)	36
Xie 2021	2016–2019	143	43	100	58.4 ± 10.6/55.4 ± 11.9	33 (76.7)/90 (90.0)	3 (7.0)/9 (9.0)	33 (76.7)/90 (90.0)	25
Squiers 2022	2014–2020	55	24	31	64.5 ± 12.8/ 64.4 ± 10.1	16 (66.7)/ 15 (48.4)	4 (16.7)/ 4 (12.9)	19 (79.2)/ 29 (93.6)	28/34
Cheng 2023	2019– 2021	93	41	52	63.0 ± 9.1/ 60.1 ± 8.9	33 (80.5)/46 (88.5)	8 (19.5)/8 (15.4)	37 (90.2)/ 45 (86.5)	13
Wu 2023	2013–2020	105	41	64	/	51 (79.7)/31 (75.6)	13 (20.3)/10 (24.4)	54 (84.3)/33 (80.5)	/

DM, diabetes mellitus; ER, endovascular revascularization; SR, surgical revascularization.

**Table 2:** Details of aortic pathology and LSA revascularisation.

Studies	Urgent intervention (ER/SR)	Aortic pathology (ER/SR)			Endovascular LSA revascularisation			Surgical LSA revascularisation	
		Aneurysm	Dissection	Other	Parallel stent	Fenestration	Single-branched stent	CSB	SCT
Bradshaw 2016	**/**	**/**	**/**	**/**	0	33	0	11	10
Zhao 2017	**/**	/	/	/	20	0	0	10	0
Piffaretti 2018	11 (36)/6 (14)	19 (61)/31 (74)	4 (13)/8 (19)	8 (26)/3 (7)	31	0	0	42	0
Xiang 2018	2 (8)/3 (21)	0 (0)/0 (0)	24 (100)/14 (100)	0 (0)/0 (0)	24	0	0	14	0
Wang 2019	0 (0)/0 (0)	0 (0)/0 (0)	17 (100)/32 (100)	0 (0)/0 (0)	0	17	0	**/**	**/**
Konstantinou 2020	0 (0)/0 (0)	10 (53)/5 (29)	6 (31.6)/12 (70.6)	3 (15.8)/0 (0)	0	19	0	15	2
D’Oria 2020	26 (22)/135 (19)	50 (43)/248 (34)	42 (36)/326 (45)	27 (21)/147 (20)	23	15	75	/	/
Johnson 2020	13 (72)/16 (46)	2 (11)/5 (14)	15 (83)/19 (54)	1 (5)/11 (31)	18	0	0	13	22
Ramdon 2020	/	6 (35)/ 45 (70)	11 (65)/19 (30)	0 (0)/0 (0)	17	0	0	64	0
Dueppers 2021	7 (29)/1 (4)	6 (25)/6 (25)	13 (54)/12 (50)	5 (21)/6 (25)	24	0	0	22	2
Xie 2021	0 (0)/5 (5)	1 (2)/5 (5)	37 (86)/84 (84)	5 (12)/11 (11)	0	43	0	100	0
Squiers 2022	0 (0)/ 3 (9.7)	11 (45.8)/12 (38.7)	12 (50)/18 (58)	1 (4.2)/1 (3.2)	0	0	24	20	11

ER, endovascular revascularization; SR, surgical revascularization; LSA, left subclavian artery; CSB, carotid-subclavian bypass; SCT, subclavian transposition.

**Figure 2:**
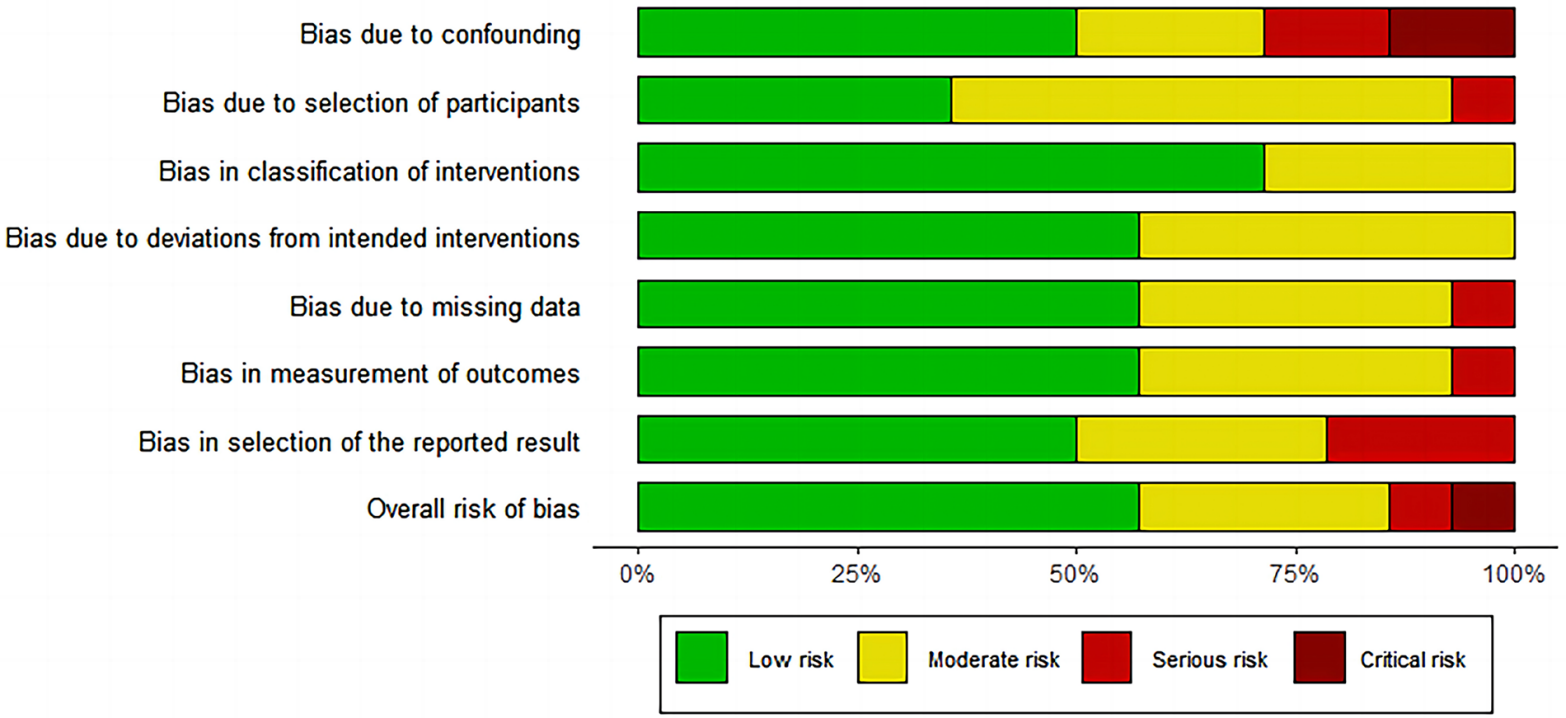
Risk of bias graph for included studies using the risk of bias in Non-randomised studies of interventions (ROBINS-I) tool.

Based on the ROBINS-I tool, we identified seven studies with low risk of bias, five with moderate risk of bias, one with serious risk of bias and one with critical risk of bias (Figure 2 and Supplementary Table S1). Six relevant outcomes were analyzed using the GRADE system. The quality of evidence was “very low” for two outcomes and “low” for four outcomes (Supplementary Table S2).

### Peri-operative stroke

**Figure 3:**
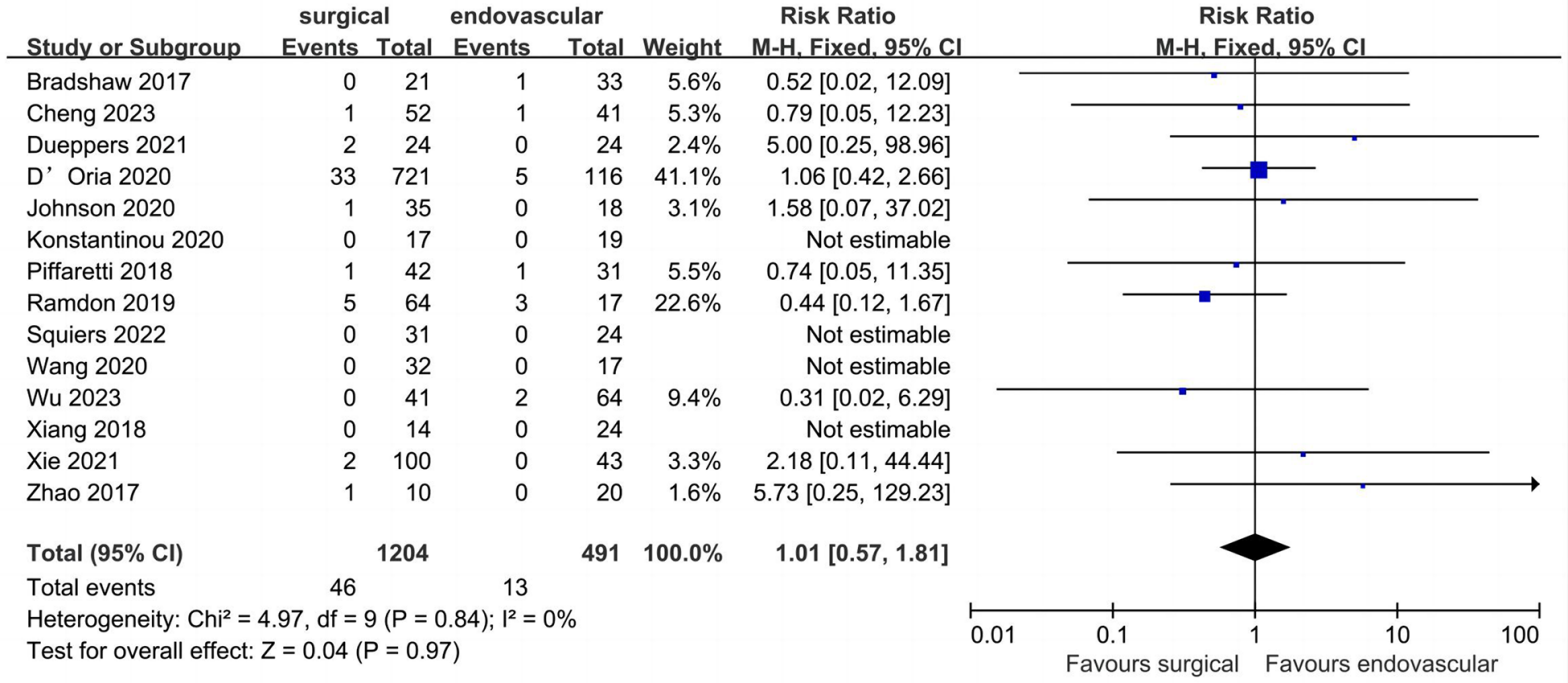
Forest plot from a fixed effects analysis of peri-operative stroke. CI, confidence interval; M-H, Mantel-Haenszel.

All included studies provided data for peri-operative stroke (Figure 3). Of the 1,695 patients included, the pooled peri-operative stroke rate was 3.4% (*n* = 1,695). The peri-operative stroke rates of the surgical and endovascular LSA revascularization groups were 3.8% and 2.6%, respectively. Compared with endovascular LSA revascularization, patients with surgical LSA revascularization showed a higher peri-operative stroke rate; however, the difference was not significant (RR: 1.01; 95% CI: 0.57, 1.81; *I*^2 ^= 0; *P* = 0.97).

### Peri-operative technical success

**Figure 4:**
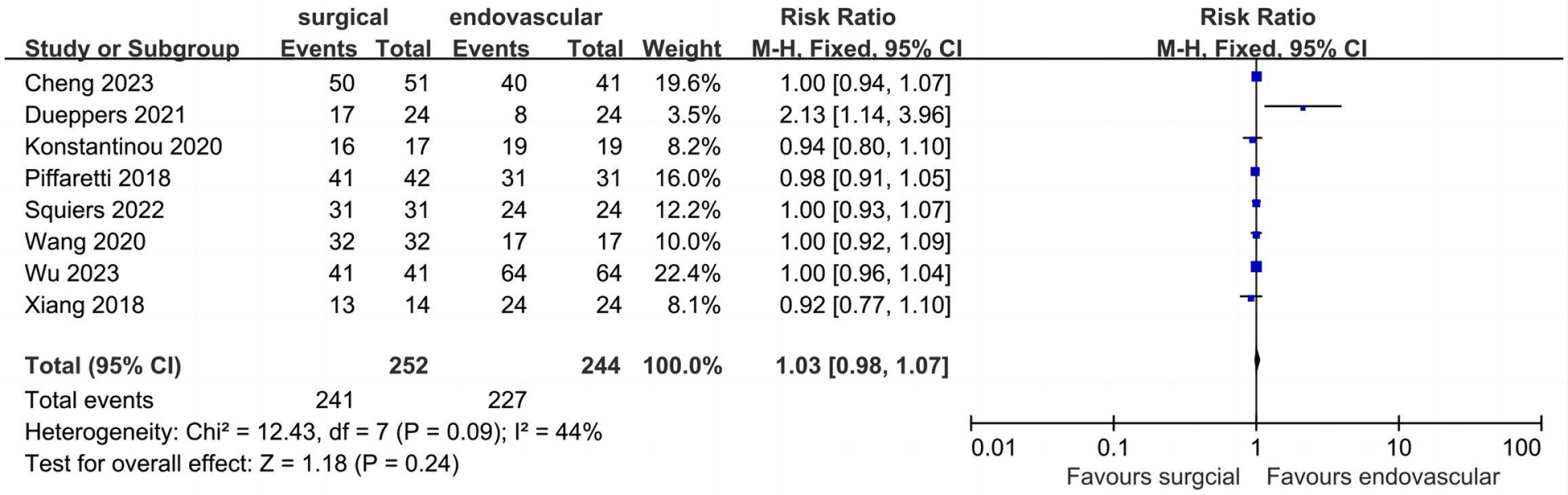
Forest plot from a fixed effects analysis of peri-operative technical success. CI, confidence interval; M-H, Mantel-Haenszel.

Eight studies described data regarding peri-operative technical success (Figure 4). Of the 496 included patients, the pooled rate of peri-operative technical success was 94.3% (*n* = 468). Peri-operative technical success rates for the surgical and endovascular LSA revascularization groups were 95.6% and 93.0%, respectively, with no significant difference observed between the groups (RR: 1.03; 95% CI: 0.98, 1.07; *I*^2 ^= 44%; *P* = 0.24).

### Peri-operative SCI

**Figure 5:**
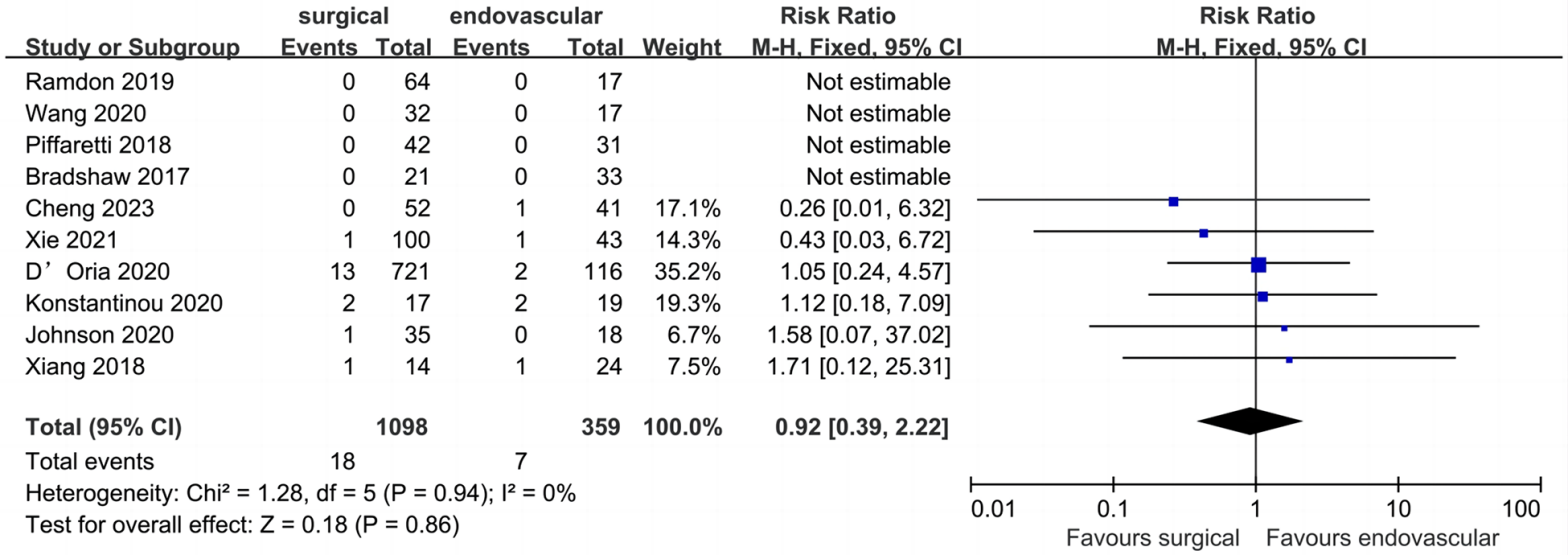
Forest plot from a fixed effects analysis of peri-operative SCI. CI, confidence interval; M-H, Mantel-Haenszel; SCI, spinal cord ischemia.

Ten studies reported data regarding peri-operative SCI (Figure 5). Of the 1457 patients included, the pooled peri-operative SCI rate was 1.7% (*n* = 25). The reported peri-operative SCI rate was 1.6% (*n* = 18) in the surgical LSA revascularization group and 1.9% (*n* = 7) in the endovascular LSA revascularization group, with no significant difference observed between the groups (RR: 0.94; 95% CI: 0.38, 2.33; *I*^2 ^= 0; *P* = 0.90).

### Peri-operative type I endoleak

**Figure 6:**
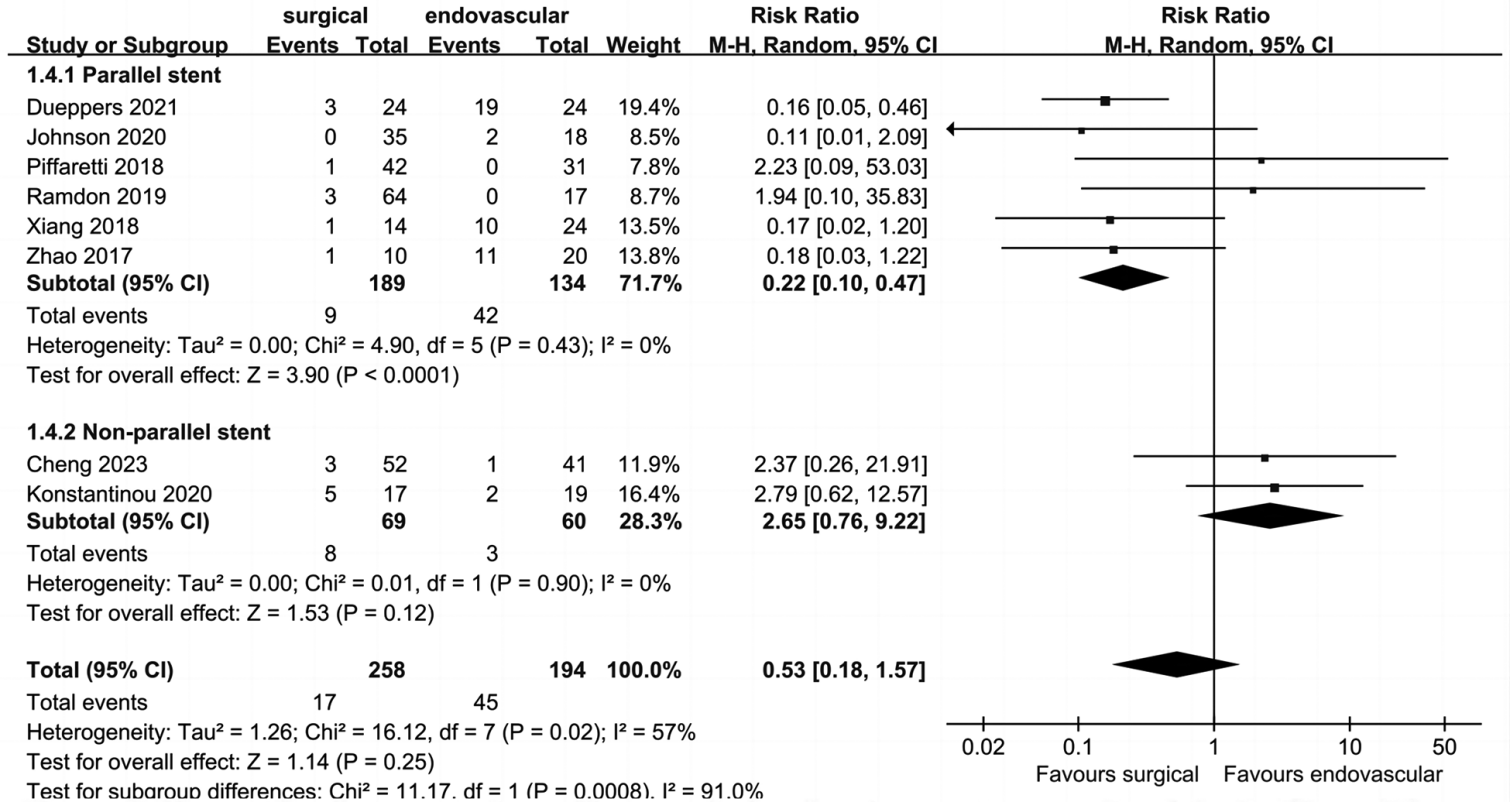
Forest plot from a random effects analysis of peri-operative type I endoleak. CI, confidence interval; M-H, Mantel-Haenszel.

Data on peri-operative type I endoleak (Figure 6) were reported in eight studies, which included 452 patients, with an overall pooled peri-operative type I endoleak rate of 13.7% (*n* = 62). Peri-operative type I endoleak rates for the surgical and endovascular LSA revascularization groups were 6.6% and 23.2%, respectively. There was no significant difference between the two groups (RR: 0.53; 95% CI: 0.18, 0.57; *I*^2 ^= 57%; *P* = 0.25).

The heterogeneity of this outcome was higher than 50%; thus, we conducted a subgroup analysis. The endovascular LSA revascularization group was divided into a parallel stent group and non-parallel stent group. The results showed that the incidence of peri-operative type I endoleak in the parallel stent group was significantly higher than that in the surgical LSA revascularization group (RR: 0.22; 95% CI: 0.10, 0.47; *I*^2 ^= 0; *P* < 0.0001). There was no significant difference between the non-parallel stent group and the surgical LSA revascularization group (RR: 2.65; 95% CI: 0.76, 9.22; *I*^2 ^= 0; *P* = 0.12).

### Peri-operative left upper limb ischemia

**Figure 7:**
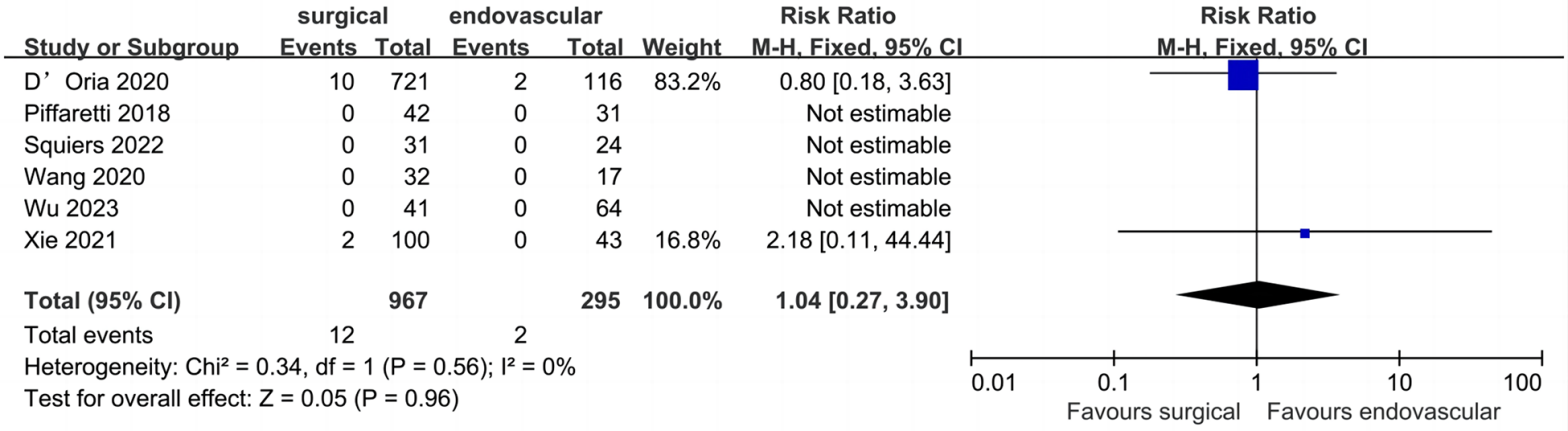
Forest plot from a fixed effects analysis of peri-operative left upper limb ischemia. CI, confidence interval; M-H, Mantel-Haenszel.

Seven studies (1,262 patients) comparing surgical LSA revascularization with endovascular LSA revascularization reported peri-operative left upper limb ischemia events (Figure 7). Of the 1,262 included patients, the pooled rate of peri-operative left upper limb ischemia was 1.1% (*n* = 14). Peri-operative left upper limb ischemia rates for the surgical and endovascular LSA revascularisation groups were 1.2% and 0.6%, respectively. No significant difference was found between the two groups (RR: 1.04; 95% CI: 0.27, 3.90; *I*^2 ^= 0; *P* = 0.96).

### Peri-operative mortality

**Figure 8:**
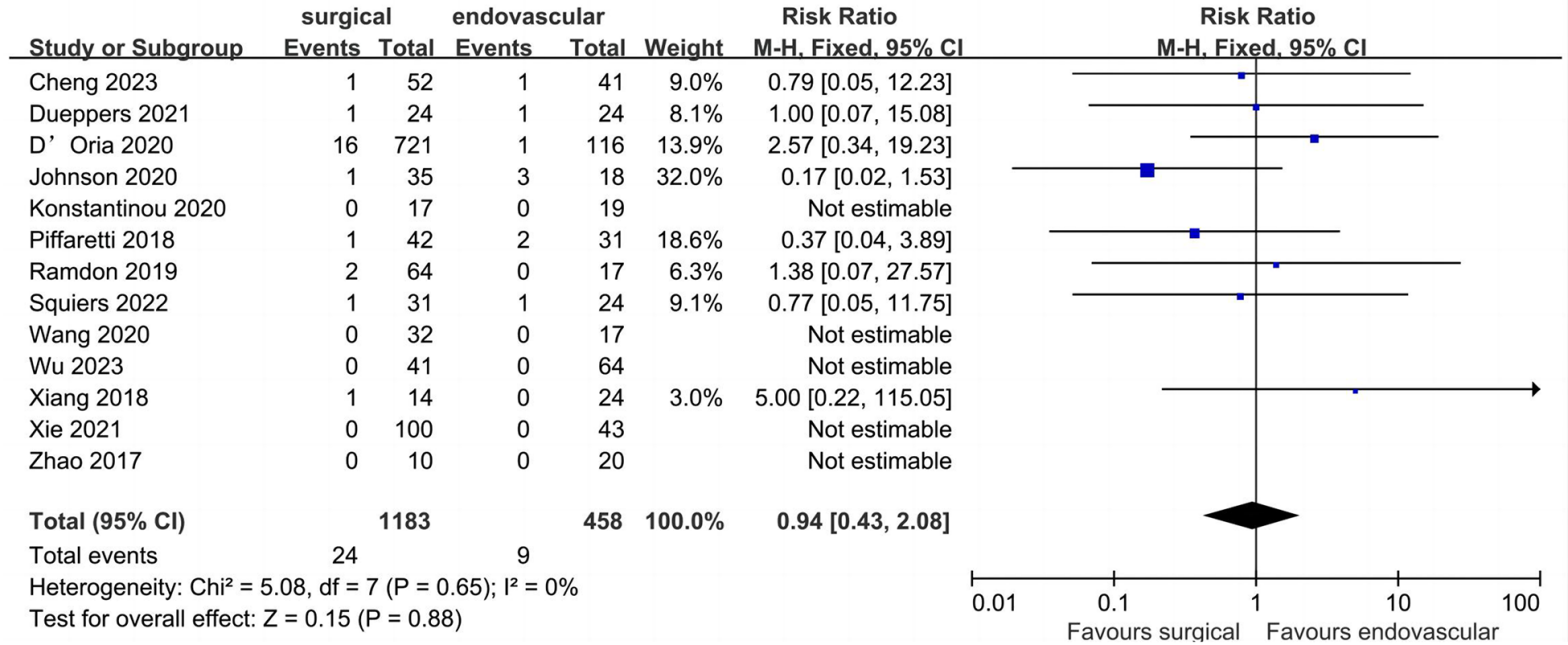
Forest plot from a fixed effects analysis of peri-operative mortality. CI, confidence interval; M-H, Mantel-Haenszel.

Thirteen studies described data regarding peri-operative mortality (Figure 8). Of the 1,641 patients included, the pooled peri-operative mortality rate was 2.0% (*n* = 33). The peri-operative mortality of the surgical and endovascular LSA revascularization groups were 2.0% and 2.0%, respectively, with no significant difference observed between the two groups (RR: 0.94; 95% CI: 0.43, 2.08; *I*^2 ^= 0; *P* = 0.88).

## Discussion

In recent years, several studies have shown that revascularization of the LSA during TEVAR could reduce complications, such as stroke and SCI (27, 28, 29). With the development of new medical devices, more medical institutions have chosen endovascular therapy to reconstruct the LSA. This systematic review and meta-analysis included 14 retrospective cohort studies (1,695 patients) and compared the difference between surgical and endovascular LSA revascularization. The pooled results showed that both treatment methods were safe and effective; there were no significant differences in the outcomes of peri-operative stroke, SCI, type I endoleak, mortality, technical success, and left upper limb ischemia.

A meta-analysis published by Hajibandeh et al. in 2016, which included five studies with a total of 1,161 patients, found that LSA revascularization did not reduce the neurological complications or mortality of patients with LSA coverage (30). However, a relatively small number of studies were included and some relevant studies were omitted. Therefore, the results of the analysis do not fully reflect real world data. Huang et al. added 11 studies in the updated meta-analysis, which included a total of 16 cohort studies (2,591 patients) (31). The pooled results showed that patients who underwent LSA revascularization had a significantly lower incidence of peri-operative stroke than patients who did not undergo LSA revascularization (5.4% vs. 7.8%, *P* = 0.001). All studies included in the present systematic review and meta-analysis were published after 2016, with peri-operative stroke rates of 3.8% in the surgical LSA revascularization group and 2.6% in the endovascular LSA revascularization group, respectively. Compared with previous studies, the overall incidence rate is still low.

Left carotid-subclavian bypass (CSB) and subclavian transposition (SCT) are traditional methods used for surgical LSA revascularization. Protack et al., from Cleveland Clinic, reported on 282 TEVAR patients who underwent 288 surgical LSA revascularization procedures, with a total of 269 (93%) CSB and 19 (7%) SCT. The incidence of 30-day stroke was 3.5%; the 1, 2, and 5-year primary patency rates were 99.5%, 98.9%, and 98.0%, respectively; and the 1, 5, and 10-year overall survival rates were 82%, 60%, and 42%, respectively. This large sample study conducted over 16 years fully demonstrated the safety and effectiveness of surgical LSA revascularization (4).

Both CSB and SCT techniques provide good patency rates; however, they were both associated with surgical and neurological complications (32, 33). A study by Konstantinou et al. included 211 patients who underwent surgical reconstruction of the LSA either before or after TEVAR. There were 24 cases (11.4%) of local bleeding and 21 cases (10.4%) of re-intervention. Local peripheral nerve injury occurred in 19 patients (9.5%), chylous fistula occurred in eight patients (3.8%), local wound infection occurred in five patients (2.4%), and one patient (0.5%) developed a bypass graft infection. The incidence of stroke was 4.3% (*n* = 9/211). The incidence of peri-operative stroke in surgical LSA revascularization in the present study was 4.0%, and the incidence of other peri-operative complications was also low. Although there was a higher trend compared to endovascular LSA revascularization, the difference was not significant.

Currently, endovascular LSA revascularization mainly includes parallel stent (chimney and periscope) techniques, fenestration techniques, and single-branched stent techniques. The parallel stent technique was first applied by Crisdo et al. in 2002 as an emergency rescue operation to save LSA blood flow during TEVAR (34). The main advantage of parallel stent technology is that it is not required to be pre-customized and can be used for immediate surgical treatment using commercial stents, resulting in it being commonly used in emergency surgery. The main problem currently affecting the widespread application of the parallel stent technique is the presence of gutter between aortic covered stents, parallel stents, and the aortic wall, leading to type I endoleak. A meta-analysis published by Ahmad et al., which included 11 studies on the use of the chimney graft technique, showed that the incidence of type Ia endoleak ranged from 0 to 20%, and the overall estimated proportion of early type Ia endoleak was 9.4% (95% CI: 6.5%, 13%) (35). Ding et al. reported the results of LSA preservation in patients with type B aortic dissection using the chimney technique; 141 patients underwent LSA revascularization using the chimney technique, and 30 patients (19%) experienced immediate type Ia endoleak (36). In the present study, the incidence of type I endoleak in the endovascular LSA revascularization group was 23.2%. Although there was no significant difference compared to the surgical LSA revascularization group, it remains a problem that deserved attention. However, the subgroup analysis of this study showed that the rate of type I endoleak in the parallel stent group was significantly higher than that in the surgical LSA revascularization group, which may limit the use of parallel stents in the LSA revascularization. In the included literatures, 20 patients in Zhao et al. ’s study received chimney stent implants (14). The 20 patients were divided into a type I endoleak group (*n* = 11) and a non-type I endoleak group (*n* = 9). The risk factors for a type I endoleak after chimney stent implantation showed that branch angulation and oversizing were important potential risk factors for postoperative type I endoleak in chimney TEVAR. Most type I endoleak occurred with large branch angles (LSA, >38.8°). In addition, the risk of an endoleak was significantly lower when oversizing of the thoracic stent graft was >11%.

Fenestration techniques include *in situ* fenestration and pre-fenestration. Mc Williams first reported the success of *in situ* fenestration in 2004 (37). In 2020, a study by Zhao et al., in China, included 130 patients who underwent *in situ* laser stent graft fenestration of the LSA during TEVAR. The 5-year follow-up results showed that the LSA patency rate was 97%, and four cases with type I endoleak disappeared during follow-up. There were no neurological complications and no deaths. These results suggest that *in situ* laser fenestration for LSA revascularization is efficient, safe, and feasible (38). Pre-fenestration included *in vitro* fenestration and custom fenestration. In 2012, a Japanese study reported the early results of a multicenter clinical trial of 383 patients with a custom fenestration stent (Najuta) for endovascular revascularization of the superior branch of the aortic arch. The technical success rate was 99.22% (*n* = 380) and the 30-day mortality rate was 1.6% (*n* = 3). Seven patients experienced cerebrovascular accident (1.8%) and permanent paralysis occurred in three patients (0.8%). Furthermore, ascending aorta dissection was observed in three patients (0.8%) (39). Fenestration with TEVAR was performed with a high success rate and low complication rate for the patients with LSA revascularization. However, the position and size design of the fenestrated stent and the precise release of the stent during the operation requires strict control by the surgeon. Furthermore, the structure of the stent was damaged by fenestration, the long-term safety needs to be further studied.

The single-branch stent technique was first proposed by Inoue et al. in 1996, and was successfully used to repair Stanford type B aortic dissection involving the LSA. Subsequently, they reported the early and mid-term results of 17 patients treated with the Inoue single-branched stent for thoracic aortic lesions involving the LSA in 2005, demonstrating the effectiveness of the Inoue single-branched stent (40, 41). A meta-analysis published in 2022 investigated the use of the Castor stent for LSA revascularization (42). The study included 11 articles with a total of 415 patients, and the pooled results showed a technical success rate of 97.5%. The early type I endoleak rate was 1.6%, the 30-day mortality rate was 0.96%, the early re-intervention rate was 0.9%, the incidence of perioperative stroke was 0%, and the 1-year survival rate was 99.7%. The results of this study showed that use of the Castor stent to reconstruct the LSA during TEVAR was feasible and effective.

The main limitations of this systematic review and meta-analysis include the following points. Firstly, the included studies were all retrospective studies; therefore, this study is lacking in RCTs. Secondly, there was a deficiency in long-term follow-up data. Thirdly, the number of articles included in this study was too small to conduct subgroup analyses. Finally, one of the reasons for the absence of a statistically significant difference between these two treatment arms could be the low number of patients in the endovascular LSA revascularization group.

## Conclusion

There was no significant difference in terms of short-term outcomes when comparing the surgical and endovascular LSA revascularization techniques. The quality of evidence assessed by GRADE scale was low to very-low. Use of both techniques during TEVAR was found to be safe and effective. Compared with surgical LSA revascularization techniques, parallel stent revascularization of the LSA significantly increased the rate of type 1 endoleak.

## Data Availability

The original contributions presented in the study are included in the article/**Supplementary Material**, further inquiries can be directed to the corresponding author/s.
