# Supplementary material for: Comparison of surgical and endovascular left subclavian artery revascularization during thoracic aortic endovascular repair: a systematic review and meta-analysis
Source: Front Cardiovasc Med. 2023 Nov 2;10:1274629. doi: 10.3389/fcvm.2023.1274629 (PMC10658894; doi:10.3389/fcvm.2023.1274629)
Supplement: Supplementary file 1 [file Table1.doc]

| Study | Confounding | Participant selection | Intervention classification | Deviations from intended intervention | Missing data | Outcome measurement:effectiveness | Outcome measurement: adverse effect | Result selection: effectiveness | Result selection: adverse effects |
| --- | --- | --- | --- | --- | --- | --- | --- | --- | --- |
| Bradshaw  2017 | Critical | Moderate | Moderate | Moderate | Moderate | Moderate | Moderate | Critical | Low |
| Cheng  2023 | Low | Moderate | Low | Low | Low | Low | Low | Low | Moderate |
| Dueppers  2021 | Low | Low | Low | Moderate | Moderate | Low | Serious | Moderate | Low |
| D’Oria  2020 | Moderate | Moderate | Low | Low | Low | Moderate | Low | Low | Low |
| Johnson  2020 | Moderate | Moderate | Low | Low | Moderate | Low | Moderate | Low | Moderate |
| Konstantinou 2020 | Low | Low | Low | Low | Low | Moderate | Low | Serious | Low |
| Piffaretti 2018 | Low | Low | Low | Moderate | Low | Low | Moderate | Low | Low |
| Ramdon  2019 | Low | Moderate | Low | Moderate | Serious | Low | Serious | Low | Moderate |
| Squiers  2022 | Low | Low | Low | Low | Low | Moderate | Low | Moderate | Low |
| Wang  2020 | Moderate | Moderate | Low | Moderate | Low | Low | Serious | Low | Serious |
| Xiang 2018 | Low | Low | Low | Low | Moderate | Low | Moderate | Moderate | Low |
| Xie 2021 | Serious | Moderate | Moderate | Moderate | Low | Low | Low | Low | Moderate |
| Zhao 2017 | Serious | Moderate | Moderate | Low | Low | Serious | Low | Moderate | Low |
| Wu 2023 | Critical | Serious | Moderate | Low | Moderate | Moderate | Low | Low | Low |

Supplementary TableS1. Risk of bias assessment using the ROBINS-I

Supplementary TableS2. GRADE assessment for each outcomes

| Outcomes | Illustrative comparative  risks* (95% CI) | | Relative  effect (95%CI) | No. of  Participants  (Studies) | Quality of the evidence  (GRADE) |
| --- | --- | --- | --- | --- | --- |
|  | Assumed risk (Endovascular) | Corresponding  risk (Surgical) |  |  |  |
| Peri-operative stroke | 26 per 1000 | 27 per 1000 | RR 1.01  (0.57 to  1.81) | 1695  (14 studies) | ⊕⊕⊝⊝  low |
| Peri-operative technical  success | 930 per 1000 | 958 per 1000  (912 to 995) | RR 1.03  (0.98 to  1.07) | 496  (8 studies) | ⊕⊝⊝⊝  very low |
| Peri-operative SCI | 19 per 1000 | 18 per 1000  (8 to 43) | RR 0.92  (0.39 to  2.22) | 1457  (10 studies) | ⊕⊕⊝⊝  low |
| Peri-operative type Ⅰ  endoleak | 232 per 1000 | 123 per 1000  (42 to 364) | RR 0.53  (0.18 to  1.57) | 452  (8 studies) | ⊕⊕⊝⊝  low |
| Peri-operative left upper  limb ischemia | 7 per 1000 | 7 per 1000  (2 to 26) | RR 1.04  (0.27 to  3.9) | 1262  (7 studies) | ⊕⊝⊝⊝  very low |
| Peri-operative mortality | 20 per 1000 | 18 per 1000  (8 to 41) | RR 0.94  (0.43 to  2.08) | 1641  (13 studies) | ⊕⊕⊝⊝  low |
| *The **corresponding risk** (and its 95% confidence interval) is based on the assumed risk in the comparison group and the **relative effect** of the intervention (and its 95% CI).  **CI:** Confidence interval; **RR:** Risk ratio;  GRADE Working Group grades of evidence  **High quality:** Further research is very unlikely to change our confidence in the estimate of effect.  **Moderate quality:** Further research is likely to have an important impact on our confidence in the estimate  of effect and may change the estimate.  **Low quality:** Further research is very likely to have an important impact on our confidence in the estimate  of effect and is likely to change the estimate.  **Very low quality:** We are very uncertain about the estimate. | | | | | |

Supplementary Appendix S1. Database specific search queries

**MEDLINE(PubMed)**

(“thoracic aortic endovascular repair” OR “TEVAR” OR “endovascular aneurysm repair” OR “endovascular stent grafting”) AND (“left subclavian artery ” OR “subclavian artery ” ) AND (“surgical revascularization” OR “endovascular revascularization” OR “transposition”)

**Embase**

(“thoracic aortic endovascular repair” OR “TEVAR” OR “endovascular aneurysm repair”) AND (“left subclavian artery” OR “subclavian artery”) AND (“surgical revascularization” OR “endovascular revascularization” OR “transposition”)

**Cochrane**

#1 MeSH descriptor: [endovascular aneurysm repair] this term only

#2 MeSH descriptor: [subclavian artery] this term only

#3 MeSH descriptor: [arteries] this term only

#4 MeSH descriptor: [endovascular procedures] this term only

#5 TEVAR

#6 surgical revascularization

#7 endovascular revascularization

#8 transposition

#9 thoracic aortic endovascular repair

#10 left subclavian artery

#11 #2 or #3

#12 #1 and (#2 or #10) and (#4 or #5)

#13 (#1 or #5) and (#6 or #7 or #8)

#14 (#1 or #5 or #7) and (#10 or #2)
